# Author Correction: Dll1+ quiescent tumor stem cells drive chemoresistance in breast cancer through NF-κB survival pathway

**DOI:** 10.1038/s41467-022-30946-9

**Published:** 2022-07-07

**Authors:** Sushil Kumar, Ajeya Nandi, Snahlata Singh, Rohan Regulapati, Ning Li, John W. Tobias, Christian W. Siebel, Mario Andres Blanco, Andres J. Klein-Szanto, Christopher Lengner, Alana L. Welm, Yibin Kang, Rumela Chakrabarti

**Affiliations:** 1 Department of Biomedical Sciences, University of Pennsylvania, Philadelphia, PA 19104 USA; 2 Department of Cancer Biology, Perelman School of Medicine, University of Pennsylvania, Philadelphia, PA 19104 USA; 3 Department of Discovery Oncology, Genentech Inc., South San Francisco, CA 94080 USA; 4 Histopathology Facility, Fox Chase Cancer Center, Philadelphia, PA USA; 5 Department of Oncological Sciences, Huntsman Cancer Institute, University of Utah, Salt Lake City, UT 84112 USA; 6 Department of Molecular Biology, Princeton University, Princeton, NJ 08544 USA

**Keywords:** Breast cancer, Cancer stem cells

Correction to: *Nature Communications* 10.1038/s41467-020-20664-5, published online 18 January 2021.

The original version of this Article contained an error in Fig. 7. In Fig. 7a panels “alphaDll1” and “IMD” were inadvertently duplicated. The correct version of Fig. 7a is shown below.
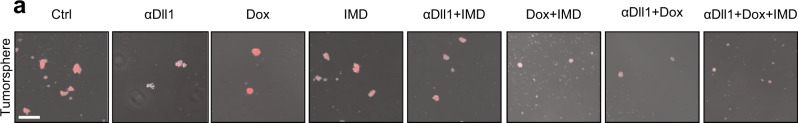


The incorrect version of Fig. 7a is shown below.
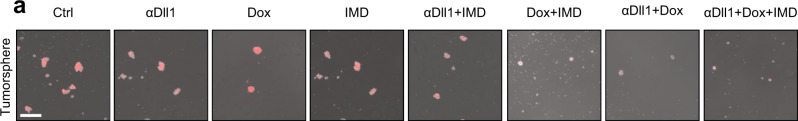


The original version of this Article contained an error in the legend of Fig. 7. The sentence “The alone doxorubicin group was used for both the experiments in Fig. 7a–b and 8 d, e (*n* = 6 Dox, *n* = 4 αDll1 ab+Dox, *n* = 4 αDll1 ab+IMD, *n* = 6 αDll1 ab+Dox+IMD tumors)” was replaced with “The alone doxorubicin and αDll1+Dox groups were used for both the experiments in Fig. 6a–b and 7d,e (*n* = 6 Dox, *n* = 4 αDll1 ab+Dox, *n* = 4 αDll1 ab+IMD, *n* = 6 αDll1 ab+Dox+IMD tumors)”.

In the original source data file of Fig. 7b, the value for cell 4f contained a wrong number.

These errors been corrected in both the PDF and HTML versions of the Article and the source data has been replaced.

