# Supplementary material for: Dll1+ quiescent tumor stem cells drive chemoresistance in breast cancer through NF-κB survival pathway
Source: Nat Commun. 2021 Jan 18;12:432. doi: 10.1038/s41467-020-20664-5 (PMC7813834; doi:10.1038/s41467-020-20664-5)
Supplement: Supplementary file 1 — Supplementary Information [file 41467_2020_20664_MOESM1_ESM.pdf]

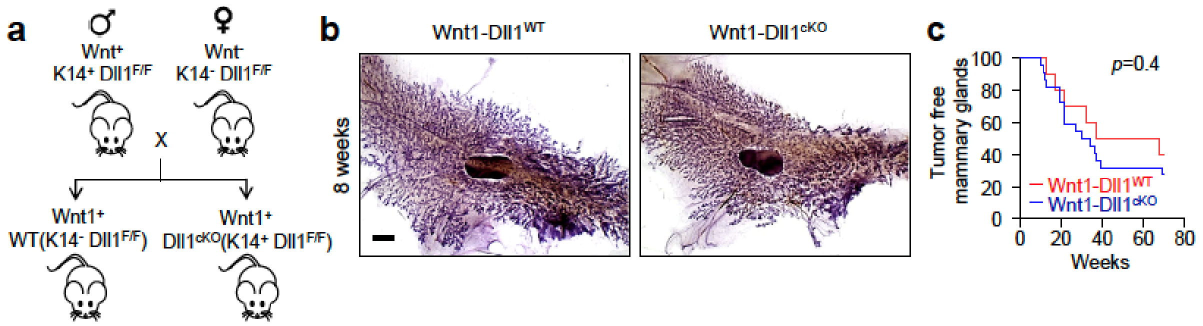

Supplementary Fig. 1: The loss of Dll1 does not influence tumor onset in the MMTV-Wnt1 model. a Schematic shows the strategy to generate MMTV-Wnt1; Dll1^{cKO} female C57BL/6 mice. **b** The representative whole mount alum carmine-stained images showed the hyperplasia stages of MMTV-Wnt1 in indicated Dll1 genotypes (n=2 mammary glands per genotype at the age of eight weeks). **c** Kinetics of mammary tumor onset in MMTV-Wnt1 females of indicated Dll1 genotypes. Wnt1-Dll1^{WT} (n=10) and Wnt1-Dll1^{cKO} (n=22). The tumors were considered positive when the tumor was 3x3 mm in dimension. P value was calculated using the two-tailed Log-Rank test (**c**). Scale bars, 500 µm (**b**). Data were obtained from two (**b**) or seven (**c**) independent experiments. Source data are provided as a source data file.

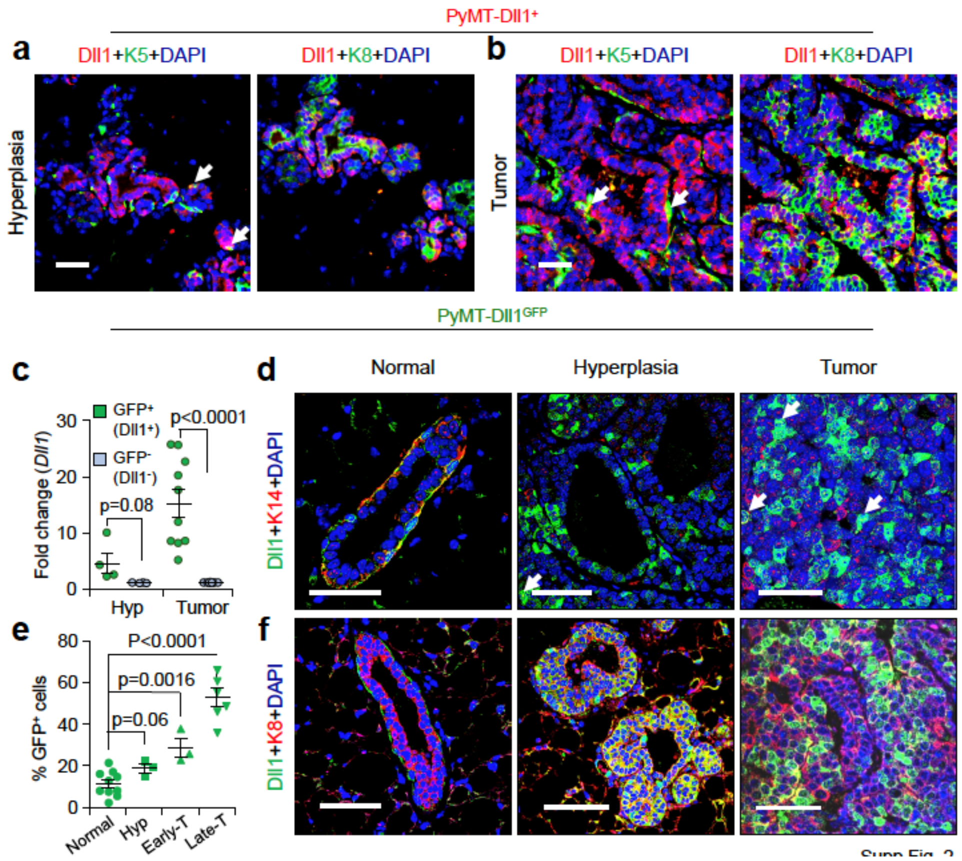

Supplementary Fig. 2: PyMT-Dll1^{mCh} and PyMT-Dll1^{GFP} reporter mice show an increased number of Dll1^{+} cells in tumors during luminal tumor development. a, b Representative immunofluorescence (IF) images of the hyperplasia and tumor of PyMT-Dll1^{mCh} mice show basal K5, luminal K8 and mCherry (Dll1) expressions (n=3 hyperplasia or tumors per group). mCherry antibody was used to detect Dll1^{mCh+} cells. White arrows indicate K5^{+}Dll1^{+} cells. **c** qPCR analyses show that Dll1^{+} (GFP^{+}) cells express a significantly higher level of *Dll1* mRNA compared to Dll1^{-} (GFP^{-}) cells. Hyperplastic mammary glands and tumors were enzymatically digested and sorted for lineage negative (CD45^{-}CD31^{-}TER119^{-}) Dll1^{+} or Dll1^{-} cells by GFP expression to quantify *Dll1* mRNA levels (n=4 hyperplasia and n=10 tumor samples). Dll1^{-} (GFP^{-}) cells were considered fold change 1. **d, f** Representative immunofluorescence (IF) images of the normal mammary

gland, hyperplasia, and tumor of PyMT-Dll1^{GFP} mice show increased number of Dll1^{+} cells with tumor development. GFP antibody was used to detect Dll1^{GFP+} expression. The Dll1^{+} cells (GFP) reside in the basal layer (K14^{+}) of the normal mammary gland, which changes during tumor progression and co-localize with luminal cells (K8^{+}) in hyperplasia and tumor (n=3 mammary gland or tumors per group). White arrows indicate K14^{+}Dll1^{+} cells. **a, b, d** White arrows indicate cells double positive for basal markers and Dll1. **a, b, d, f** Please see more representative images in the supplementary figure 11. **e** The quantification of flow cytometry data of Lin^{-} tumor cells show increasing Dll1^{+} cells by GFP expression in PyMT-Dll1^{GFP} mice from normal to Hyperplasia (Hyp) and tumor (T) stages (**e** n=10 normal, n=3 hyperplasia, n=3 early tumors and n=6 late tumors). **c** P values were calculated using two-tailed Student's *t*-test by comparing individual groups. **e** P values were calculated using One-way ANOVA with Tukey's multiple comparisons post hoc test. **c,e** Data are presented as the mean ± SEM. Scale bars, 40 μm (**a,b,d,f**). Source data are provided as a source data file.

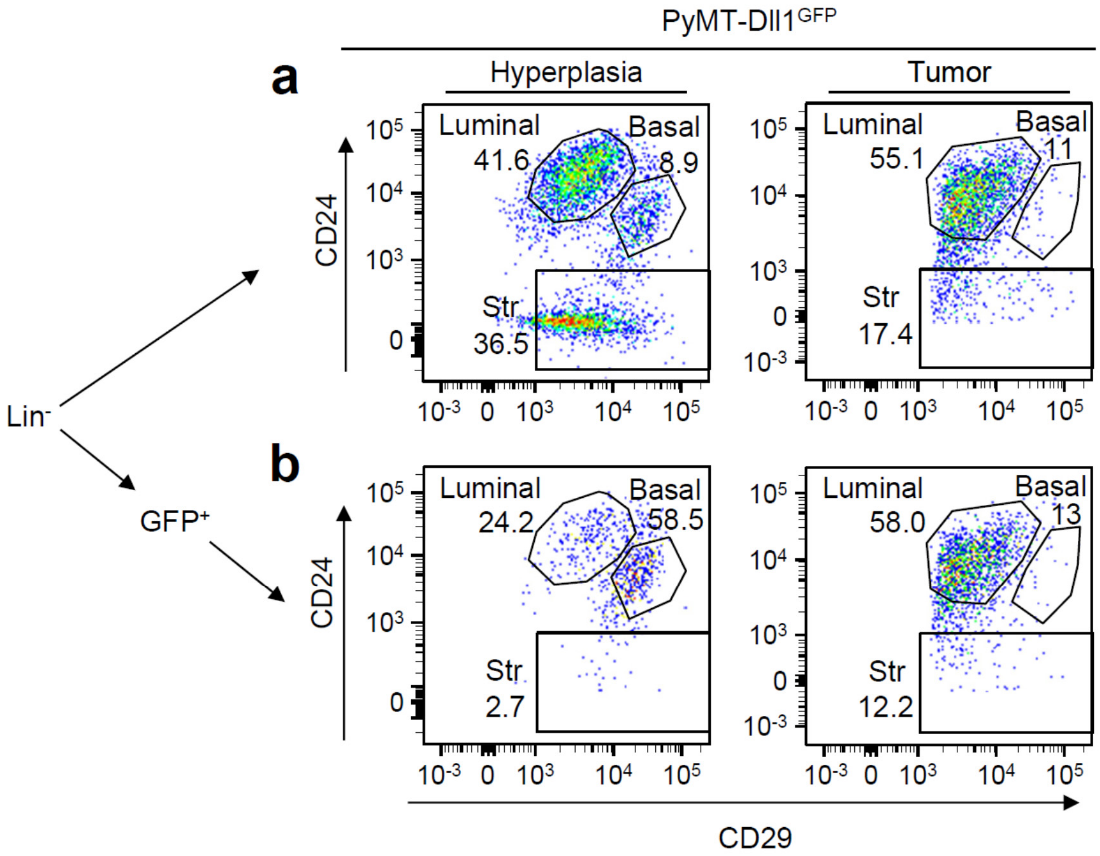

Supplementary Fig. 3: Flow cytometry data show total tumor cell population in hyperplasia and in tumor stage. a,**b** Flow cytometry profile of Lin$^-$ cells (**a**) or Lin$^-$ Dll1$^+$ cells (**b**) from PyMT-Dll1^{GFP} hyperplasia and tumor show basal and luminal population during luminal tumor progression. As hyperplasia progresses to tumor, more luminal cells are observed in the tumors. n=3 PyMT-Dll1^{GFP} hyperplasia (at age of 8-weeks) and tumors. Seven independent experiments were performed to obtain the represented flow figures.

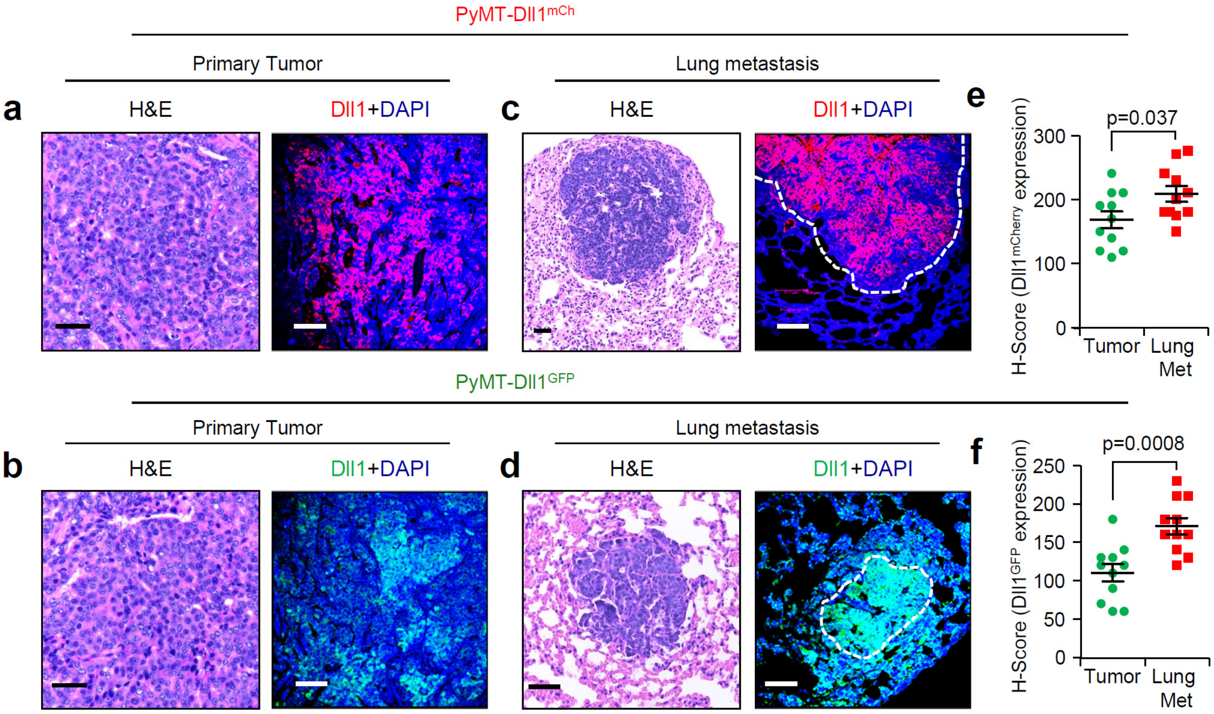

Supplementary Fig. 4: Dll1 is strongly expressed in metastatic lung tumor cells compared to primary mammary tumor. a,b Representative tumor H&Es show tumor morphology and IF images show expression of Dll1 in the primary tumors of PyMT-Dll1^{mCh} (**a**) and PyMT-Dll1^{GFP} mice (**b**). **c,d** Representative H&Es depict lung nodule morphology and IF images show expression of Dll1 in metastatic lung nodules of PyMT-Dll1^{mCh} (**c**) and PyMT-Dll1^{GFP} mice (**d**). mCherry antibody was used to detect Dll1^{mCh+} cells in tumor or lung nodules of PyMT-Dll1^{mCh} mice. GFP antibody was used to detect Dll1^{GFP+} cells in tumor or lung nodules of PyMT-Dll1^{GFP}mice. **e**,**f** The scatter plots show quantification of Dll1 H-score in tumor and lung nodules of PyMT-Dll1^{mCh} (**e**) and PyMT-Dll1^{GFP} (**f**). The H-score represents the Dll1 (mCherry/GFP) intensity (scale 0-3) multiplied with abundance of Dll1^{+} cells (scale 0-100). **e**,**f** The dots in scatter plots represent n=11 FOV/group from four tumors and four lung nodules each group. For IF,

the formalin-fixed tumors and lungs were stained with the mCherry and GFP antibodies to detect Dll1^{+} tumor cells. Scale bars, 60 µm. Data present seven (**a-d**) independent experiments. Data are presented as the mean ± SEM. Seven independent experiments were performed to obtain the represented IF and H&E figures. **e,f** P values were calculated using two-tailed unpaired Student's *t*-test. Source data are provided as a source data file.

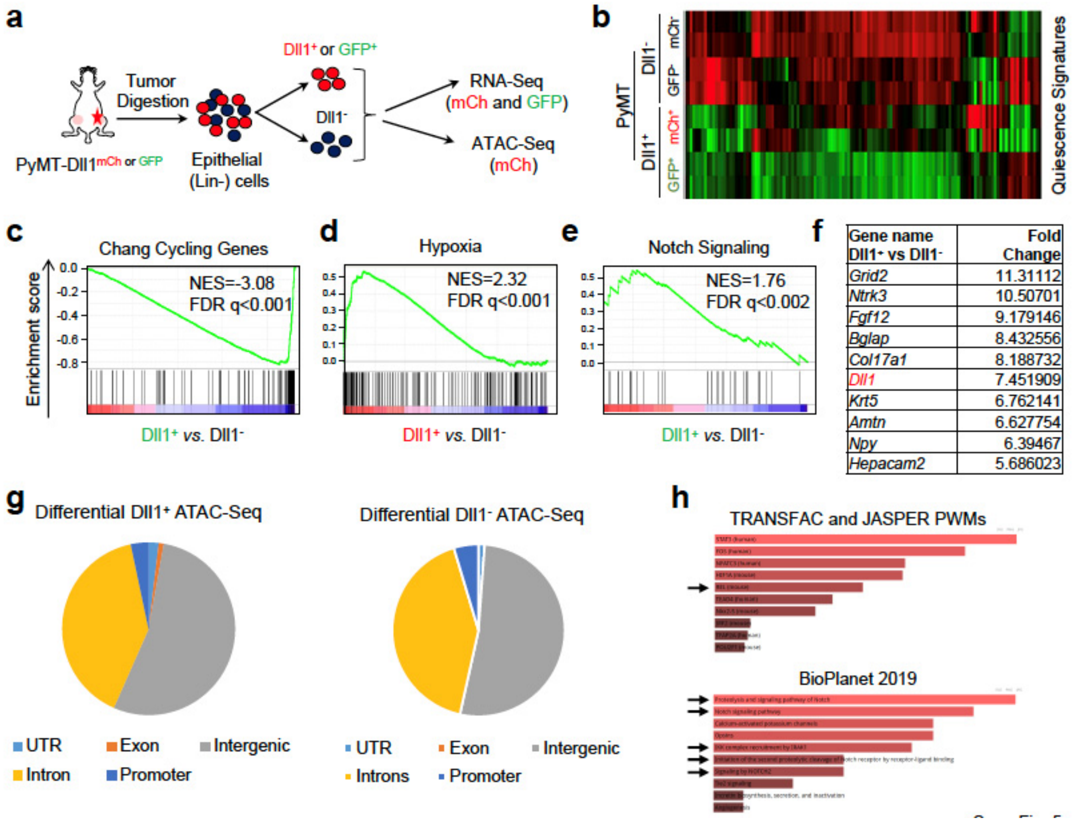

Supplementary Fig. 5: RNA-seq and ATAC-seq suggest that Dll1⁺ tumor cells are quiescent and express pro-tumoral signatures. a Schematic diagram represents the experimental strategy to perform RNA-seq and ATAC-seq using Dll1$^{+/-}$ tumor cells from PyMT-Dll1^{mCh} and PyMT-Dll1^{GFP} tumor. **b** The heat map shows the enrichment of quiescence signatures (Chang Cycling gene signature from GSEA C2 set) in Dll1^{+} tumor cells compared with Dll1^{-} tumor cells isolated from PyMT-Dll1^{mCh} or PyMT-Dll1^{GFP} tumors. **c,d, e** GSEAs of RNA-seq demonstrate decreased cycling genes or increased hypoxia and Notch pathway signatures in Lin^{-} Dll1^{+} tumor cells compared to Dll1^{-} tumor cells isolated from PyMT-Dll1^{mCh} (red) and PyMT-Dll1^{GFP} (green) tumors, respectively. **f** 10-metastasis gene classifier was identified using stringent cut off of +5.5 fold change with a p-value less than 0.05 by combining PyMT-Dll1^{mCh} and PyMT-Dll1^{GFP} reporter mice's

RNA-seq data. **g** The pie charts show the chromatin accessibility in $Dll1^+$ and $Dll1^-$ tumor cells, indicating no dramatic differences. **h** EnrichR software was used to show enrichment of core 23 UP genes of GSEA (from Fig. 4e) for Notch and NF-κB pathway from combined ATAC-seq and RNA-seq analyses. PyMT-$Dll1^{mCh}$ model was used for both RNA-seq and ATAC-seq whereas PyMT-$Dll1^{GFP}$ was used only for RNA-seq (n=2 samples for $Dll1^+$ and n=2 samples for $Dll1^-$ Lin^- tumor was used from PyMT-$Dll1^{mCh}$ tumors for RNA-seq; n=2 samples for $Dll1^+$ and n=2 samples for $Dll1^-$ Lin^- tumor cells was used from PyMT-$Dll1^{GFP}$ tumors for RNA-seq; and n=4 $Dll1^-$ and n=3 $Dll1^+$ Lin^- tumor cells isolated from PyMT-$Dll1^{mCh}$ tumors for ATAC-seq).

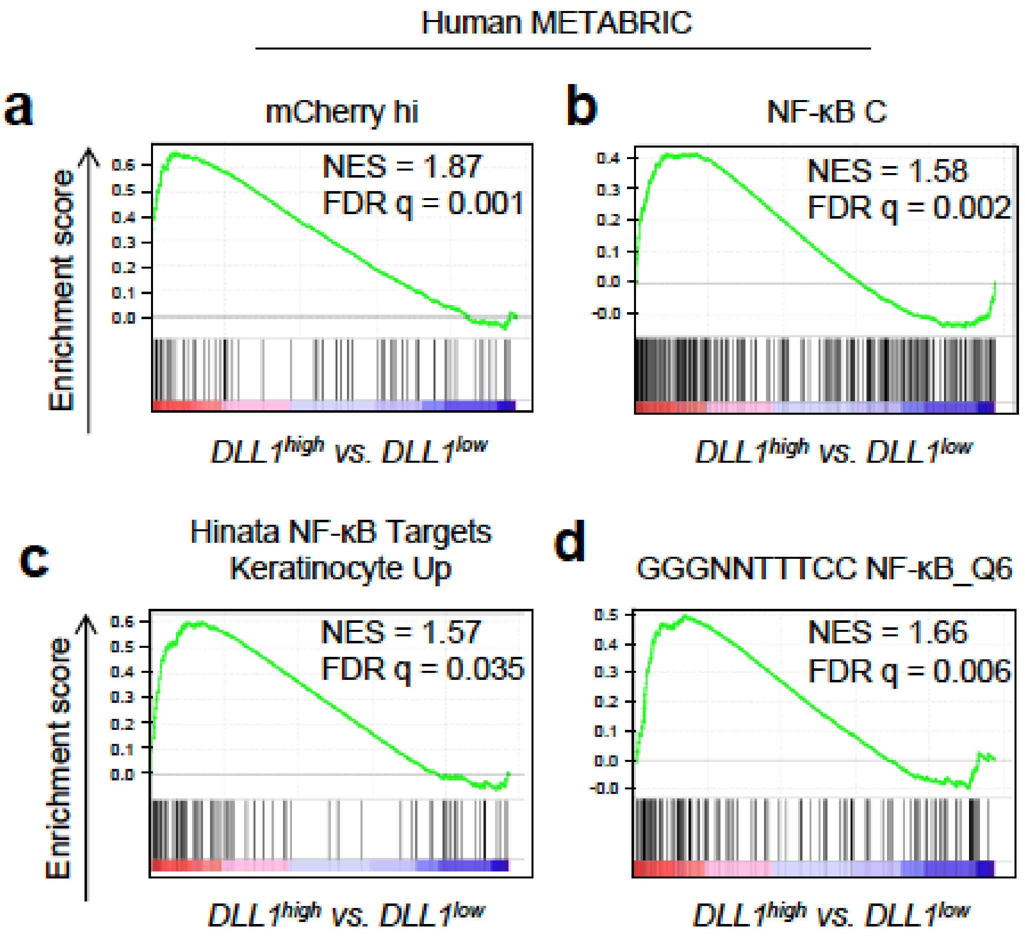

Supplementary Fig. 6: Patient data show upregulation of multiple NF-κB signatures in Dll1^{high} patient tumors. a GSEA depicts high enrichment of Dll1^{+} tumor cells signatures from PyMT^{mcherry} to patient tumors (Luminal A and B) with high *DLL1* expression. **b-d** GSEAs show various increased NF-κB signatures in Luminal A and B patient tumors after stratifying them on *DLL1* levels. n=1140 patient tumors. The patient data was obtained from METABRIC dataset[1,2].

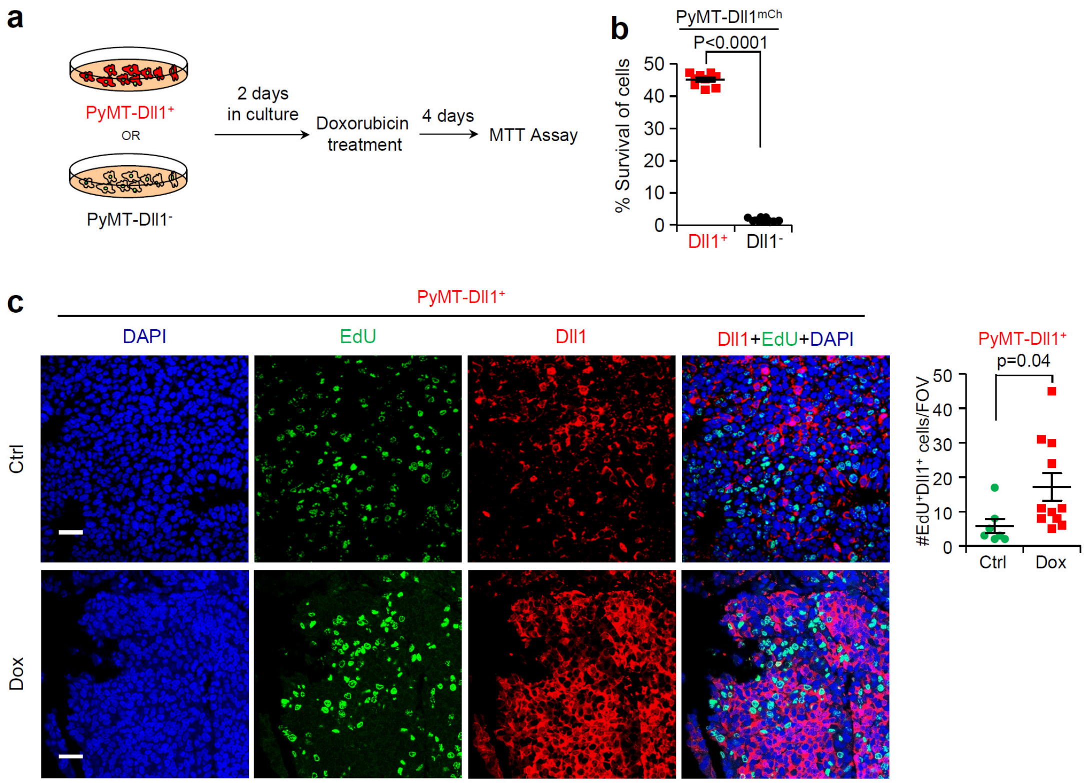

Supplementary Fig. 7: Dll1^{+} tumor cells are less-proliferating and chemotherapy-resistant. **a** Schematic shows the experimental plan to perform *in vitro* drug-sensitive assay with Dll1^{+} and Dll1^{-} tumors using 100ng/mL dose of Doxorubicin. **b** MTT assay results show the doxorubicin-resistant characteristics of PyMT-Dll1^{+} tumor cells compared with PyMT-Dll1^{-} cells. Sorted Dll1^{+} and Dll1^{-} tumor cells were seeded in 96-well plate (n=9 wells each) for MTT assay. **c** The single color and merged IF images show the expression of Dll1 (red) and EdU^{+} proliferating cells (green) in PyMT-Dll1^{+} tumors upon treatment of saline or doxorubicin. mCherry antibody was used to detect Dll1^{mCh+} cells. The number of double positive (Dll1^{+} and EdU^{+}) cells were counted in the right. The dots in scatter plot represents the multiple field of views (indicated in image), which is obtained from n=3 tumors per group. Scale bars, 40 μm. Data are presented as the

mean ± SEM. P values were calculated using two-tailed unpaired Student's *t*-test. FOV is field of view. Source data are provided as a source data file.

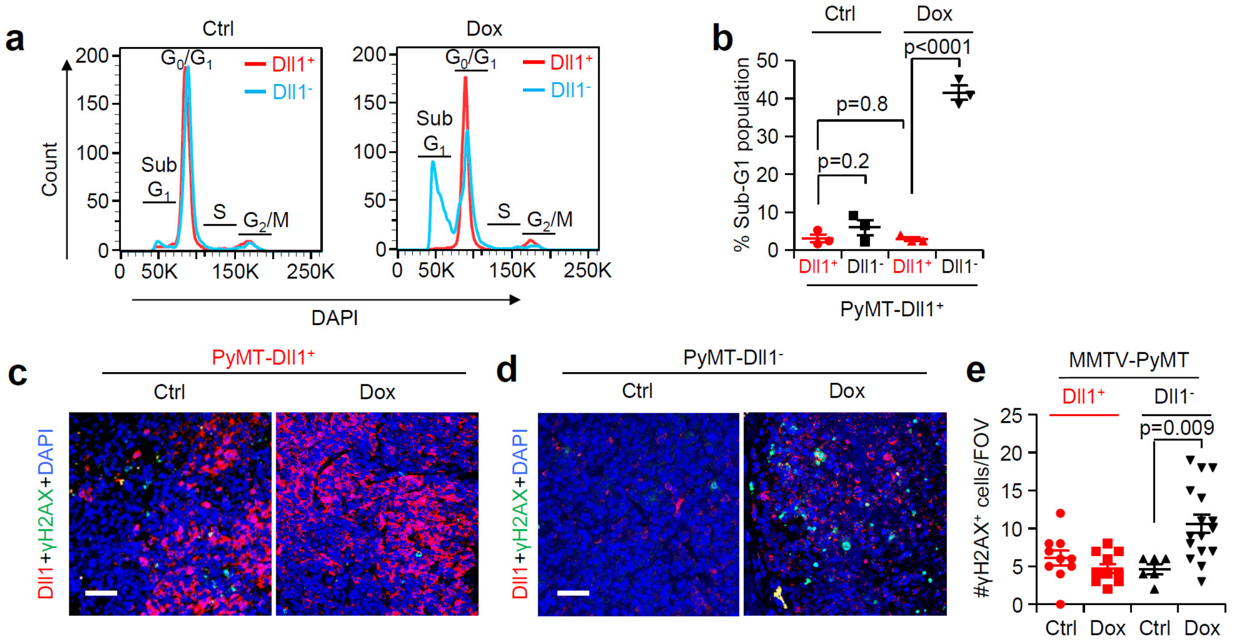

Supplementary Fig. 8: Dll1⁺ tumor cells escape apoptosis. a,b Cell cycle analyses of PyMT-Dll1⁺ tumor cells indicate a significant increase in the Sub-G1 population of Dll1⁻ tumor cells (blue line) compared with Dll1⁺ tumor cells (red line) upon doxorubicin treatment. The Dll1⁺ and Dll1⁻ tumor cells were separated based on the mCherry expression and percentages of apoptotic (Sub-G1) tumor cells were quantified in **b**. n=3 tumors for each group were analyzed for cell cycle by flow cytometry. **c,d** The representative IF images depict the γH2AX⁺ DNA damage in PyMT-Dll1⁺ (**c**) and PyMT-Dll1⁻ (**d**) tumors upon saline or doxorubicin treatments, which are quantified in **e**. The formalin-fixed tumors specimens were stained with the mCherry antibody to detect Dll1⁺ tumor cells. The dots in scatter plot represent multiple field of views (indicated in figure) from n=3 tumors/group and the experiment was repeated three independent times. **b,e** P values were calculated using One-way ANOVA with Tukey's multiple-comparisons post hoc test. **b,e** Data are presented as the mean ± SEM. FOV is field of view. Scale bars, 40 µm (**c, d**). Source data are provided as a source data file.

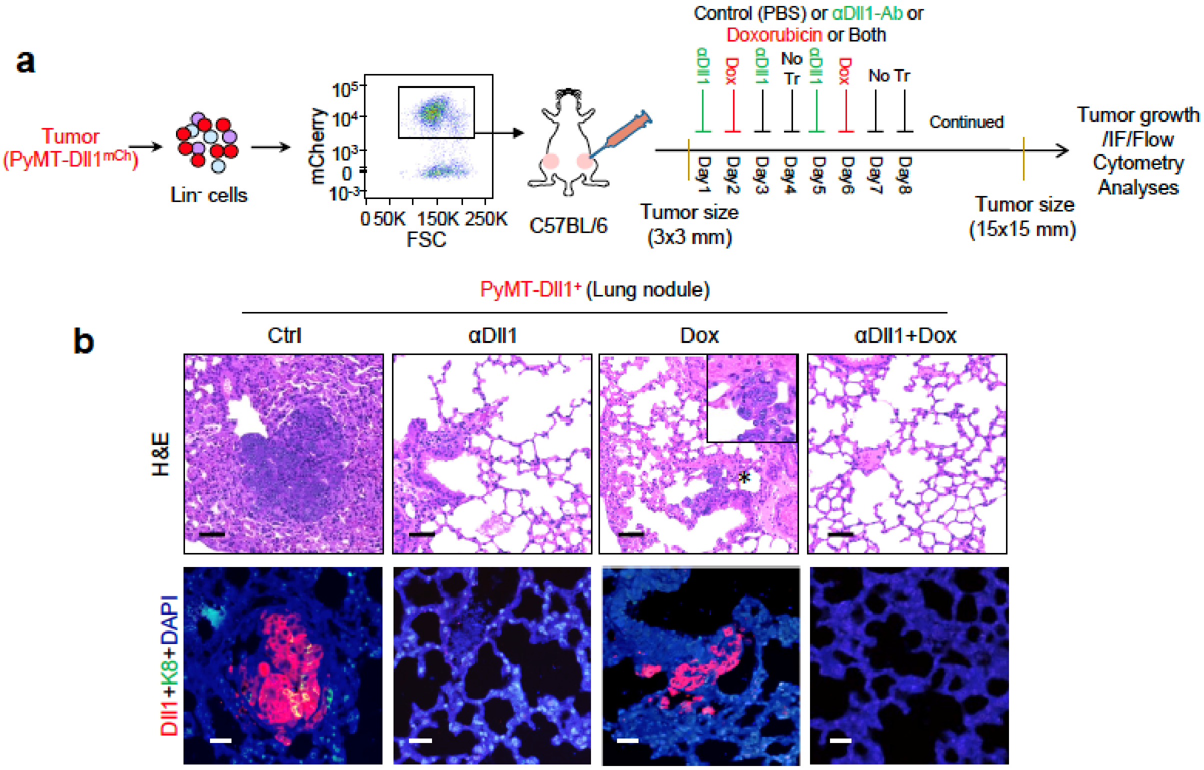

Supplementary Fig. 9: Dll1-blocking antibody inhibits metastasis of PyMT-Dll1^{mCh} bearing mice. a We transplanted sorted PyMT-Dll1^{+} tumor cells into the mammary fat pad (MFP) of syngeneic C57BL/6 mice. The schematic diagram shows the experimental plan of *in vivo* treatment of Dll1^{+} tumors with and without Dll1 blocking antibody (18mg/kg thrice a week) and doxorubicin (Dox, 1mg/kg twice a week). Control mice were treated with Saline. **b** Representative H&E and IF images show Dll1 (mCherry) positive lung metastatic nodules in tumor-bearing mice from transplanted PyMT-Dll1^{+} tumor cells (data combined from two independent experiments, n=5 Ctrl, n=5 αDll1 ab, n=6 Dox and n=5

αDll1 ab + Dox). mCherry antibody was used to detect Dll1^{mCh+} cells. **b** Inset shows zoomed in area of lung metastasis. Scale bars, 20 μm (**b**).

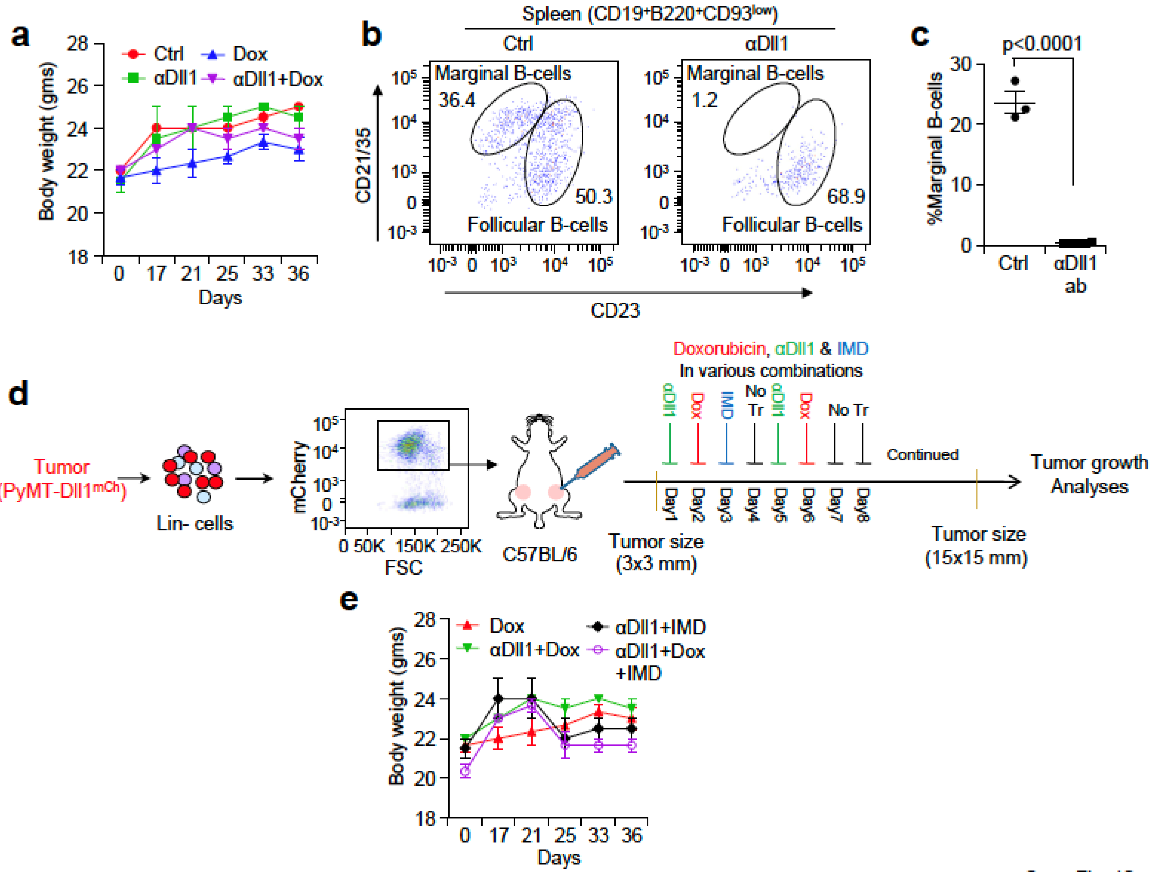

Supplementary Fig. 10: Dll1-blocking antibody has no dramatic side effects on mice health. We transplanted sorted PyMT-Dll1^{+} tumor cells into the mammary fat pad (MFP) of syngeneic C57BL/6 mice. We treated PyMT-Dll1^{+} tumors with and without Dll1 blocking antibody (18mg/kg thrice a week) and doxorubicin (1mg/kg twice a week). Control mice were treated with Saline. **a** Whole-body weight of mice with the treatment shows no significant toxicity after the indicated treatments. n=2 Ctrl, n=2 αDll1 ab, n=3 Dox and n=2 αDll1 ab + Dox mice and each mouse had two contralateral mammary gland

tumors. **b,c** Representative flow cytometry analysis (**b**) shows the effectiveness of Dll1-blocking antibody treatment by depletion of marginal B-cell population of the spleen, which is quantified in **c**. Marginal B-cells decrease in spleen of treated mice (n=3 spleens for ctrl, n=6 spleens for αDll1 ab treated groups (n=3 alone αDll1 ab and n=3 spleens for αDll1+Dox). **d** We transplanted sorted PyMT-Dll1^{+} tumor cells into the mammary fat pad (MFP) of syngeneic C57BL/6 mice. The schematic diagram shows the experimental plan of *in vivo* treatment of Dll1^{+} tumors with and without Dll1 blocking antibody (18mg/kg thrice a week), doxorubicin (1mg/kg twice a week) and IMD (0.5mg/kg once a week). Control mice were treated with Saline. **e** Whole-body weight of mice with the treatment shows no significant toxicity after the indicated treatments. n=3 Dox, n=2 αDll1 ab+Dox, n=2 αDll1 ab+IMD, n=3 αDll1 ab+Dox+IMD mice and each mouse had two contralateral mammary gland tumors. P values were calculated using two-way ANOVA with Bonferroni post-test adjustment (**a,e**) and two-tailed unpaired Student's *t*-test (**c**). **a,c,e** Data are presented as the mean ± SEM. Source data are provided as a source data file.

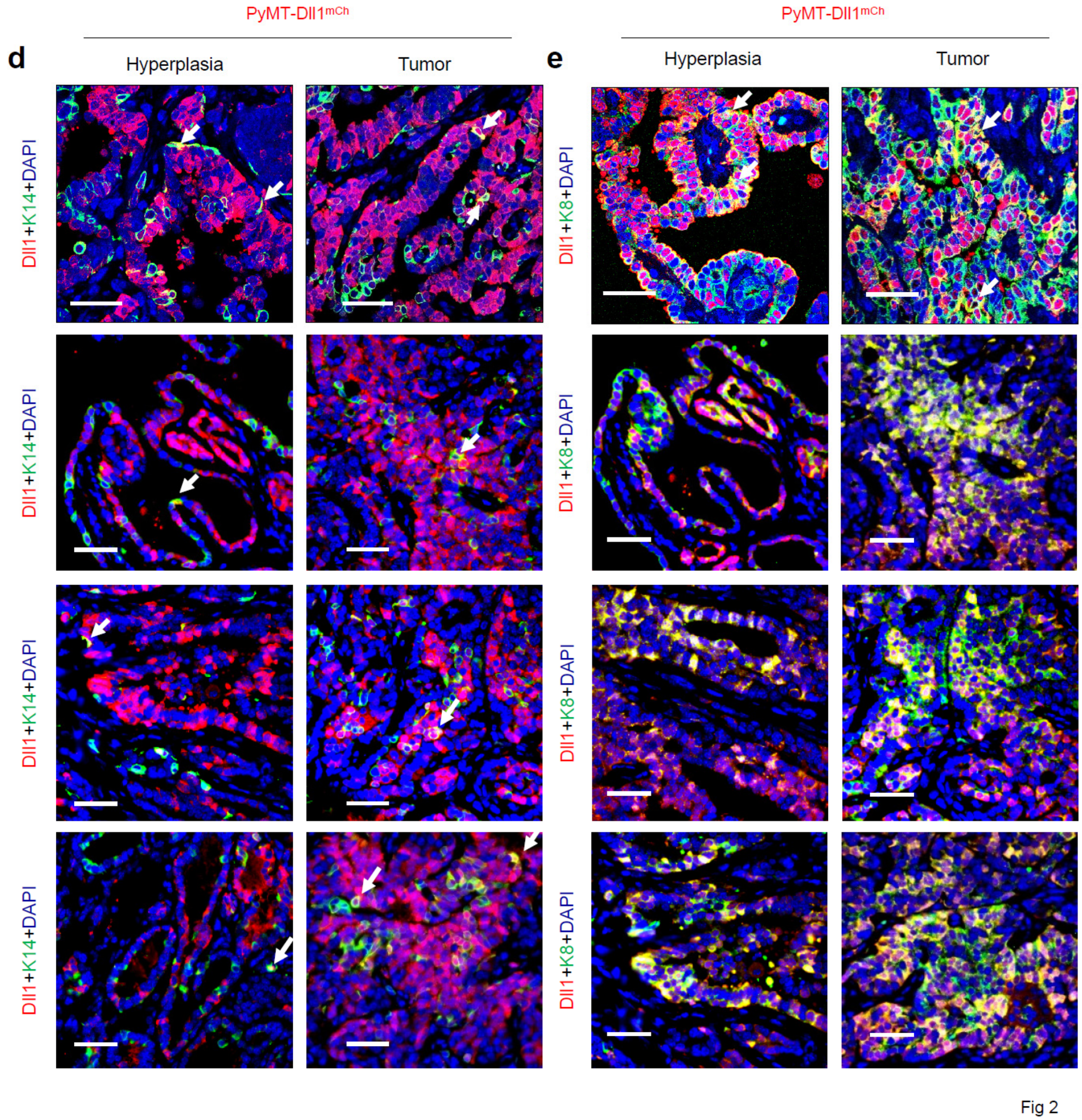

Supplementary Fig. 11: Representative IF images from Figure 2**d** and **e**. **d** White arrows indicate co-localization of basal marker K14 and Dll1. **e** White arrows indicate the co-localization of Dll1 and K8 (n=3 tumors per group). Scale bars, 60 μm.

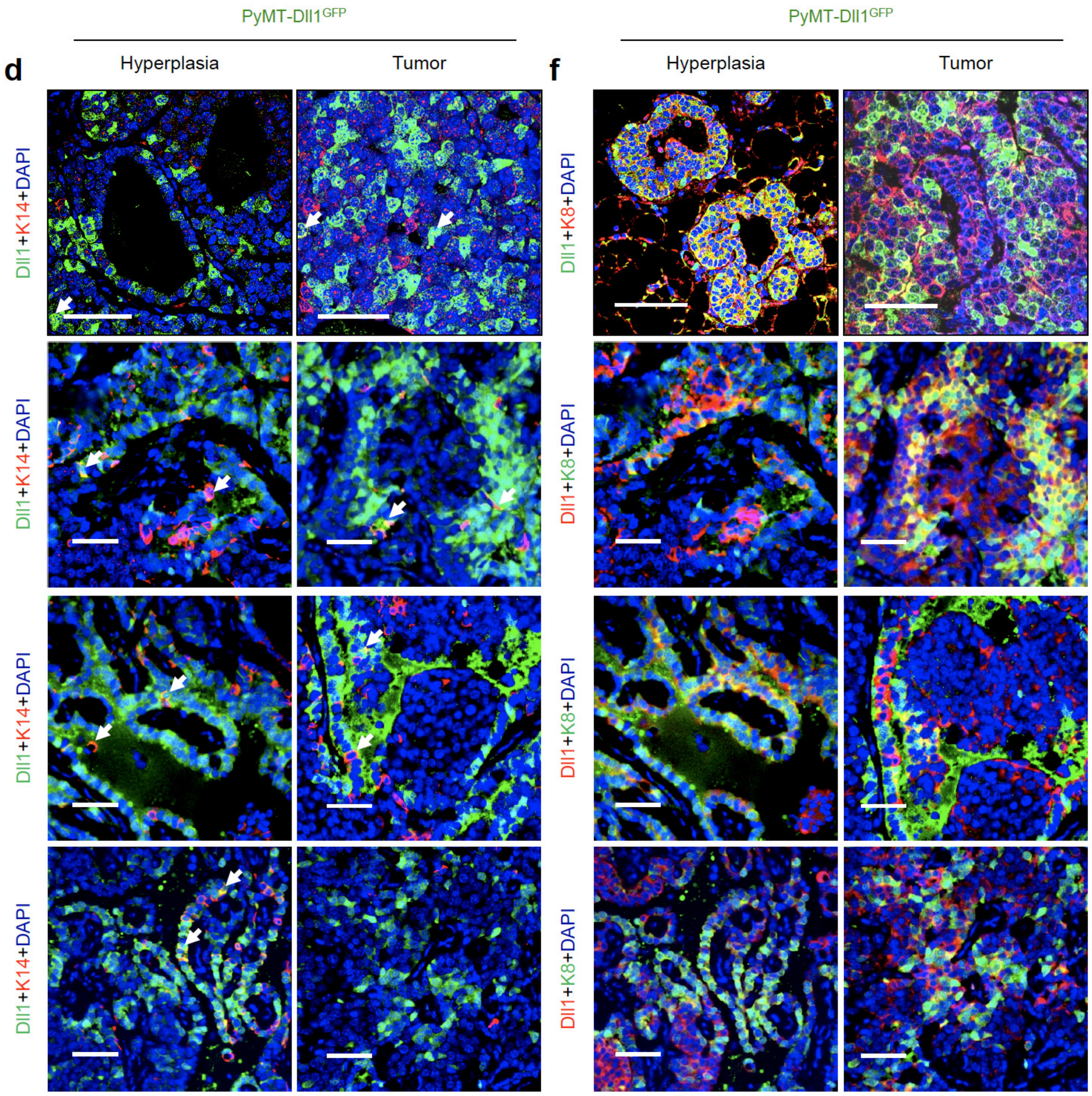

Supplementary Fig. 11: Representative IF images from Supplementary Figure 2d and f. White arrows indicate double positive cells for basal marker, K14 and Dll1. Scale bars, 60 µm.

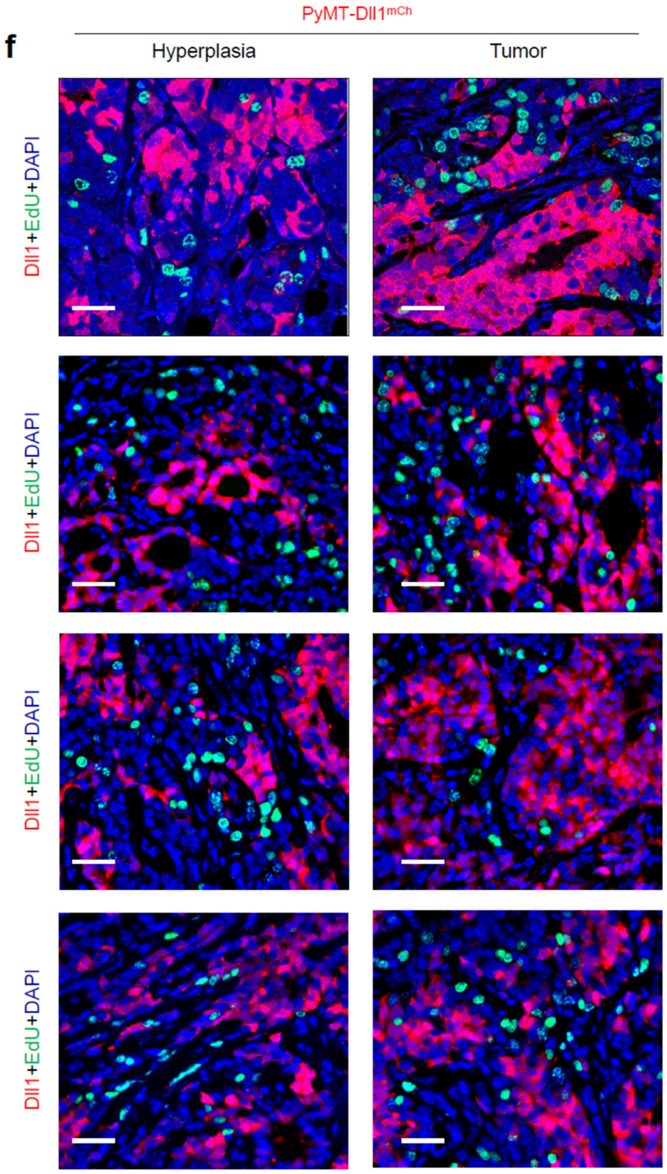

Supplementary Fig. 11: Representative IF images from Figure 3f (n=3 tumors per group). Scale bars, 60 µm.

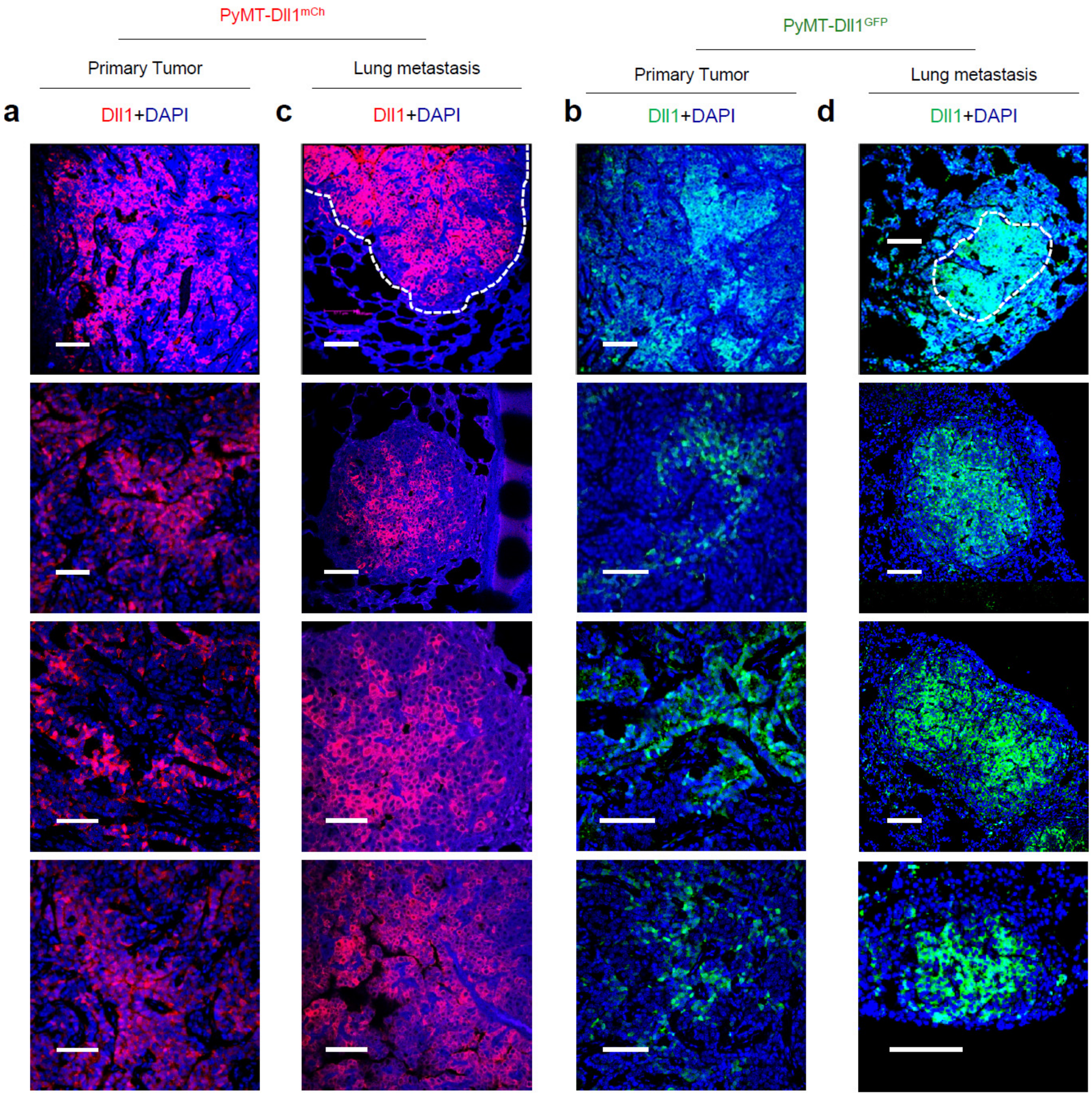

Supplementary Fig. 11: Representative IF images from Supplementary Fig. 4a-d. n>3 tumors or lung nodules per panel. Scale bars, 60 µm.

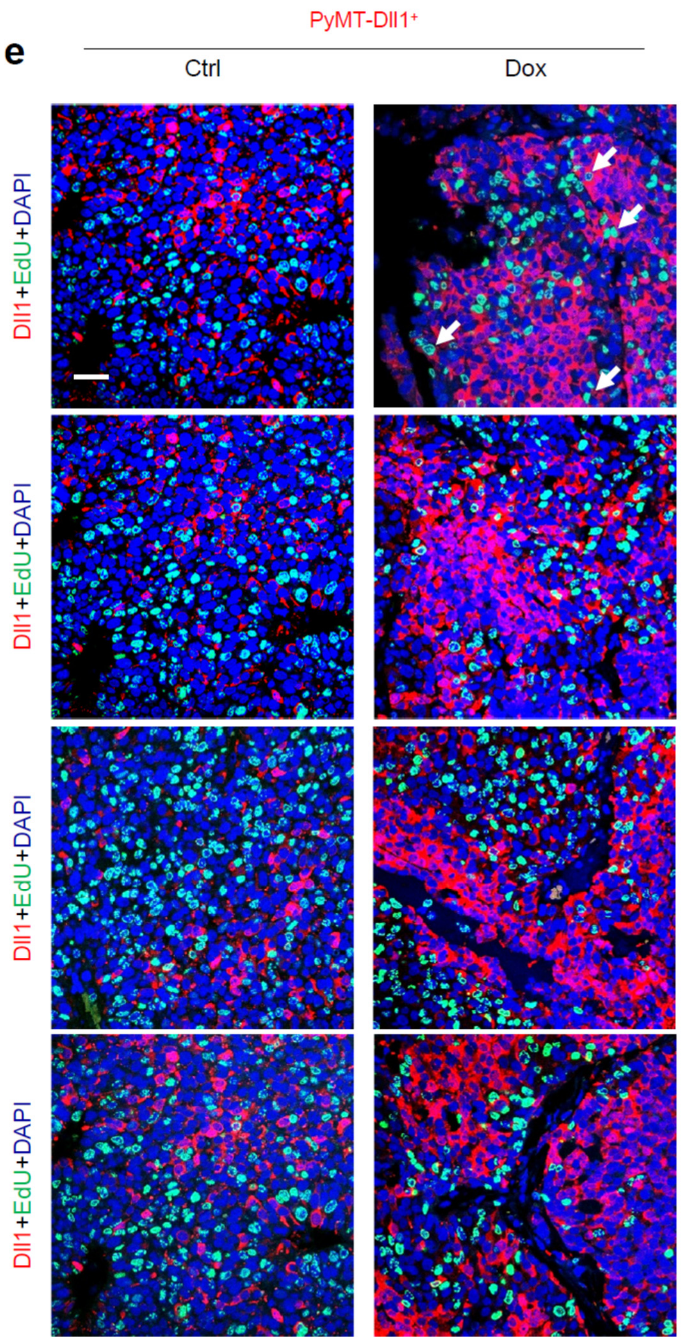

Supplementary Fig. 11: Representative IF images from Figure 5e (n=3 tumors per group). The white arrows indicate proliferating Dll1^{+} EdU^{+} tumor cells upon doxorubicin treatment. Scale bars, 60 µm.

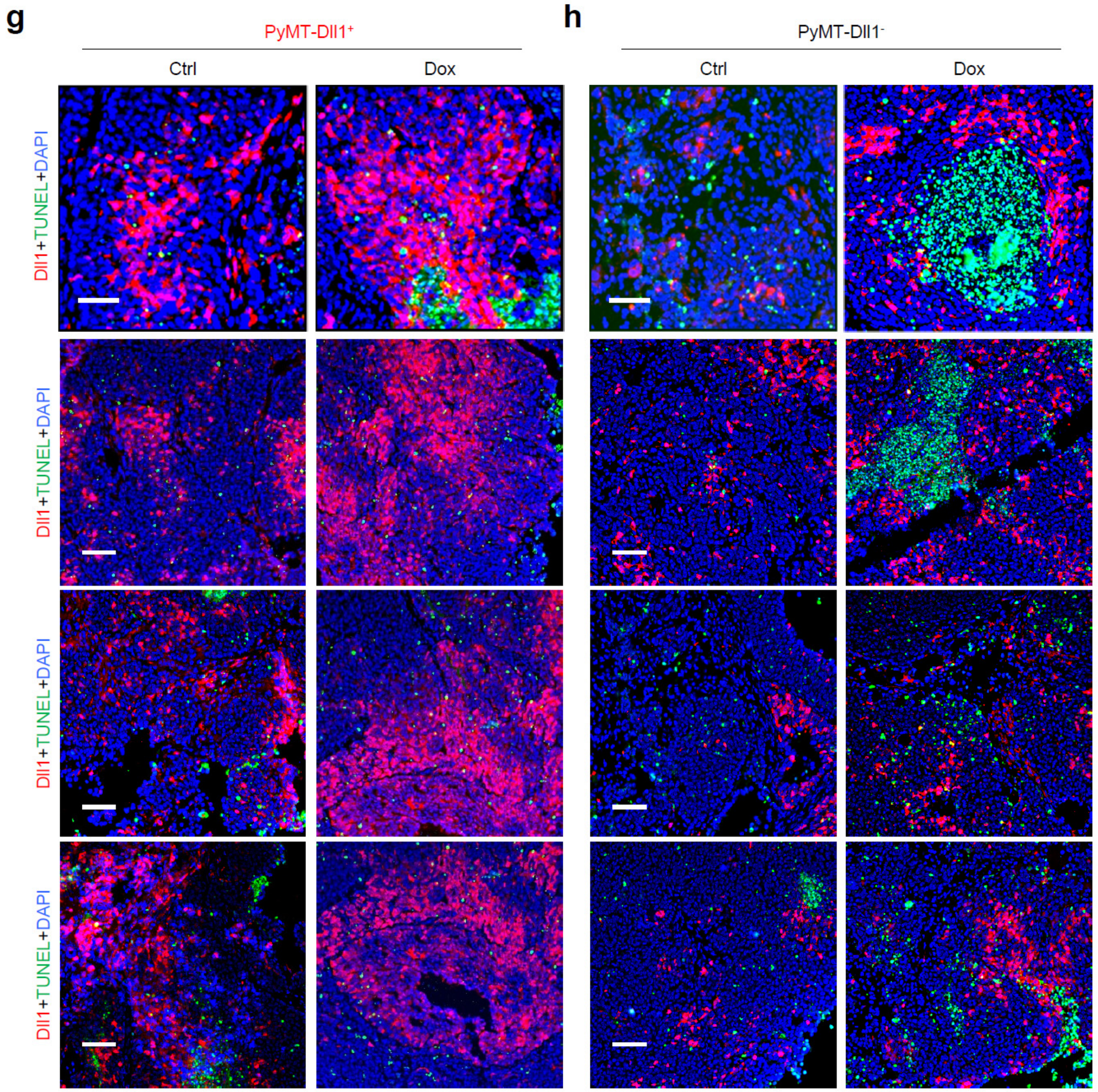

Supplementary Fig. 11: Representative IF images from Figure 5g,h (n=4 tumors per group). Scale bars, 60 μm.

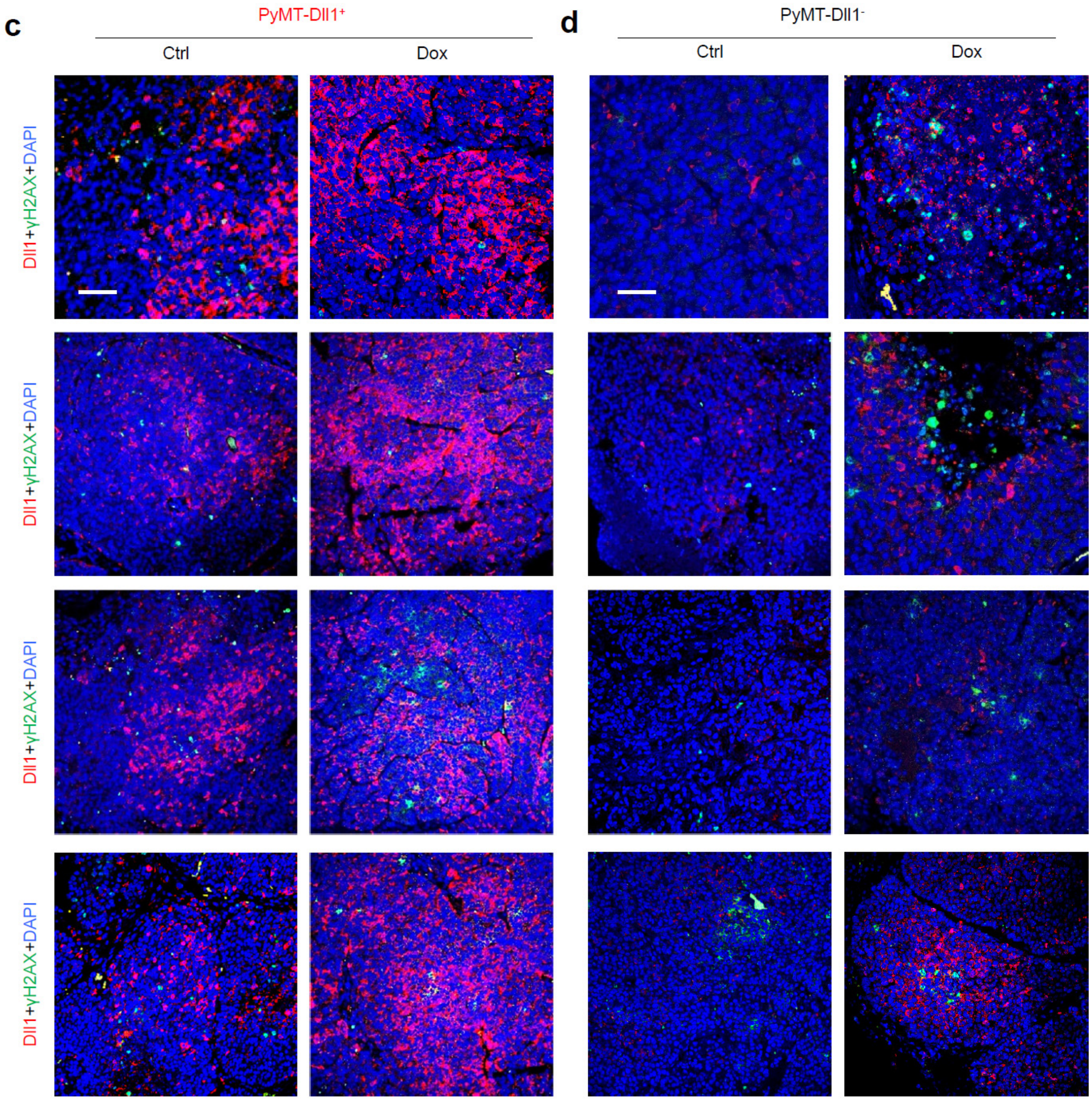

Supplementary Fig. 11: Representative IF images from Supplementary Figure 8c-d (n=3 tumors per group). Scale bars, 40 μm.

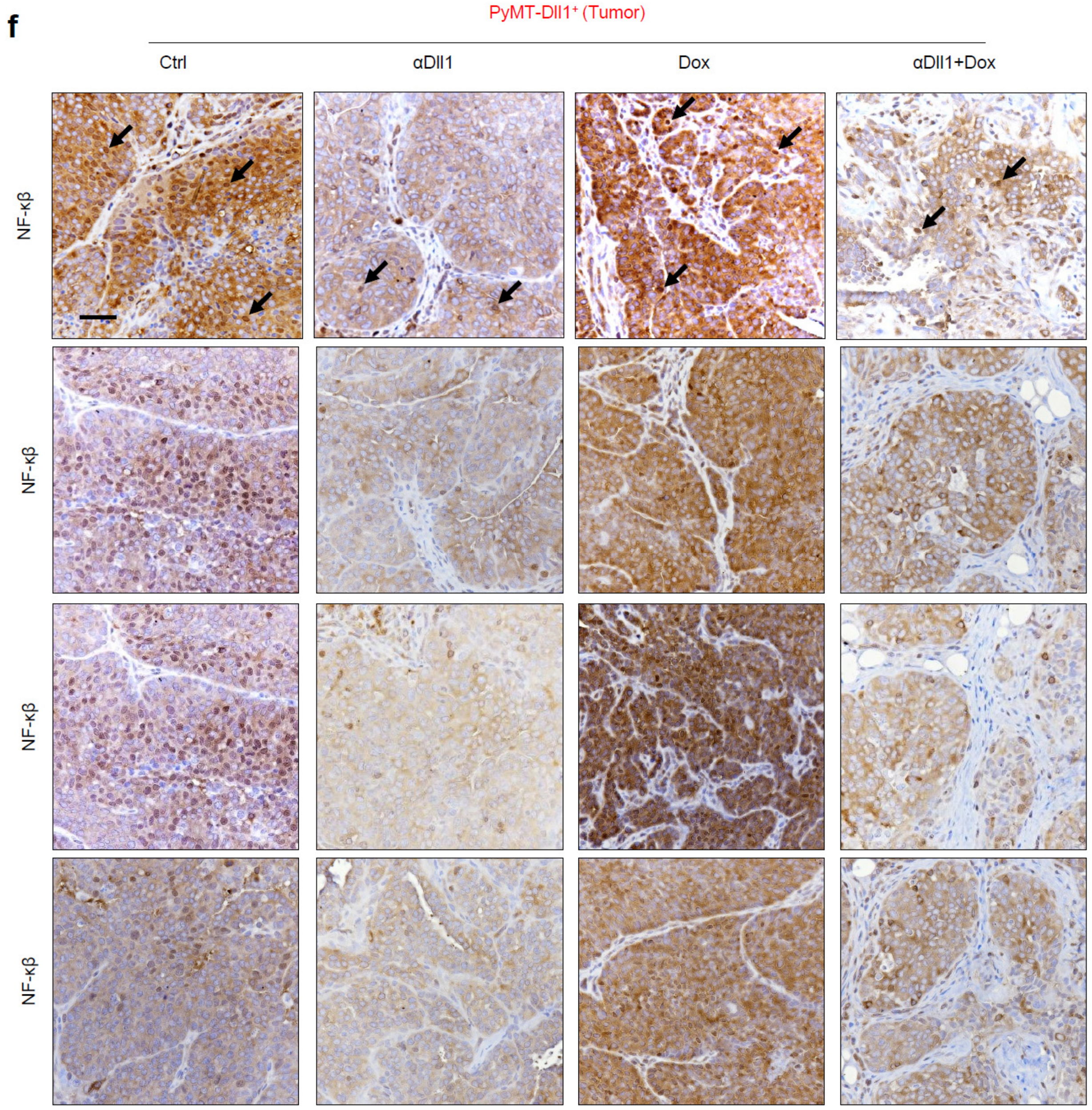

Supplementary Fig. 11: Representative IHC images from Figure 6f (n=3 tumors per group). The black arrows indicate nuclear translocation of NF-κB. Scale bars, 60 μm.

Source file for western blot from Fig.1b.

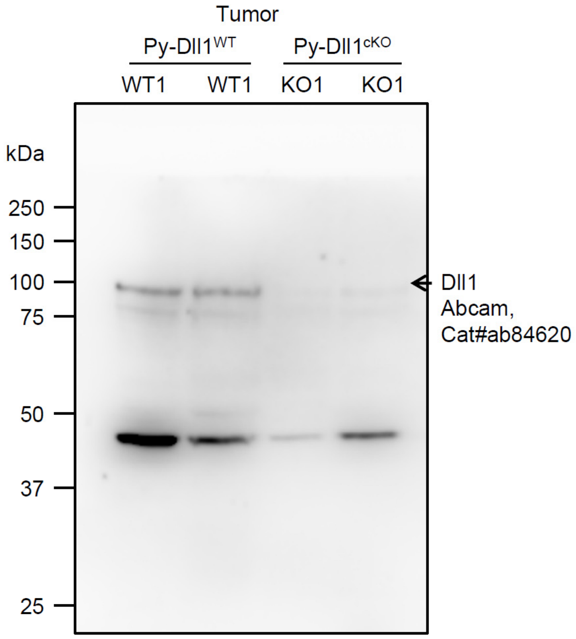

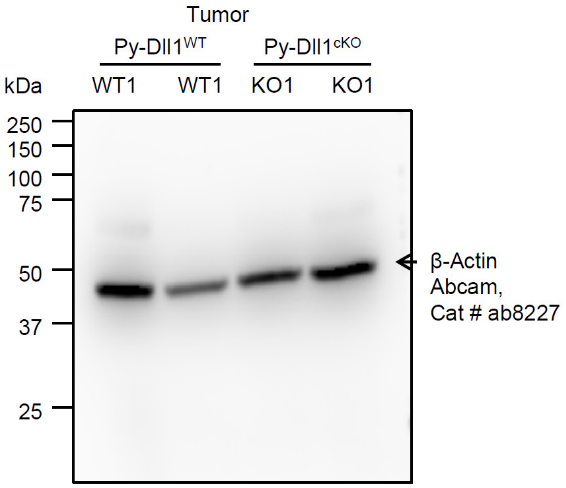

Full scan western blot images related to the figure 1b.

Supplementary Table 1: Antibody used for flow cytometry

Antibody	Fluorochrome	Dilution	Catalog #	Company
CD24	PE	1:100	553262	BD Biosciences
CD24	BV421	1:100	562563	BD Biosciences
CD29	FITC	1:100	MCA2298	Serotec/Bio-Rad
CD31	Biotin	1:100	558737	BD Biosciences
CD45	Biotin	1:100	553078	BD Biosciences
Ter-119	Biotin	1:100	553672	BD Biosciences
Streptavidin	PE-Cy7	1:150	SA1012	Invitrogen
CD19	Bv785	1:100	115543	BioLegend
CD45R/B220	APC	1:100	103212	BioLegend
CD93 (AA4.1)	PE-Cy7	1:100	136506	BioLegend
CD23	PE	1:100	553139	BD Biosciences
CD21/35	APC-Cy7	1:100	123418	BioLegend

Supplementary Table 2: Antibody used for IF and IHC

Antibodies	IF	IHC	Company and catalog number
K14 (rabbit)	1:100	-	BioLegend, Cat # 905304
K14 (chicken)	1:100	-	BioLegend, Cat# 906001
K14 (new rabbit)	1:75	-	Sinobiological, Cat# 100129-RP02
K5 (rabbit)	1:40	-	Abcam, Cat# ab52635
K8	1:100	-	Developmental Studies Hybridoma Bank, TROMA-1s
GFP	1:200	-	Abcam, Cat # ab13970
mCherry (rabbit)	1:100	-	Abcam, Cat # ab167453
mCherry (mouse)	1:100	-	Abcam, Cat # ab125096
γH2AX	1:100	-	Abcam, Cat # ab26350
NF-κB (RelA)	-	1:200	Cell Signaling, Cat# 8242
dsRed	1:100	-	Living Colors, Cat# 632392

Supplementary Table 3: Antibody used for western blot

Antibodies	Western blot	Company and catalog number
Dll1	1:1000	Abcam, Cat # ab84620
β-Actin	1:10,000	Abcam, Cat # ab8227

Supplementary Table 4: Primers used for qRT-PCR

Gene	Species	Forward	Reverse
Dll1	Mouse	GCGAGCTGCACGGACCTTGA	GCCCAAGGGGCAATGGCAGG
Gapdh	Mouse	CCCCAATGTGTCCGTCGTG	GCCTGCTTCACCACCTTCT

Supplementary Table 5: Reagent and Resource

Reagent/Kit	Source	Identifier
Tamoxifen	Sigma-Aldrich	Cat# T5648
IMD	Tocris	Cat# 2611
Purelink RNA mini kit	Thermofisher	Cat#12183018A
Rabbit Anti-caspase 3, Active	BD Biosciences	Cat#550480
Click-iT Plus EdU Alexa Fluor 594	Thermo Fisher Scientific	Cat#C10639
Click-iT™ Plus TUNEL Assay for In Situ Apoptosis Detection Kit, Alexa Fluor 488 dye	Thermo Fisher Scientific	Cat#C10617
MTT Reagent	Sigma-Aldrich	Cat#M5655
B27 Supplement (50X), Serum-free	Thermo Fisher Scientific	Cat#17504044
Recombinant Human Epidermal Growth Factor/EGF	Novoprotein	Cat#C029
Recombinant Human Fibroblast Growth Factor 2/FGF-2	Novoprotein	Cat#C046

1 Curtis, C. *et al.* The genomic and transcriptomic architecture of 2,000 breast tumours reveals novel subgroups. *Nature* **486**, 346-352, doi:10.1038/nature10983 (2012).

2 Pereira, B. *et al.* The somatic mutation profiles of 2,433 breast cancers refines their genomic and transcriptomic landscapes. *Nature communications* **7**, 11479, doi:10.1038/ncomms11479 (2016).
