# Supplementary material for: Dll1+ quiescent tumor stem cells drive chemoresistance in breast cancer through NF-κB survival pathway
Source: Nat Commun. 2021 Jan 18;12:432. doi: 10.1038/s41467-020-20664-5 (PMC7813834; doi:10.1038/s41467-020-20664-5)
Supplement: Supplementary file 3 — Reporting Summary [file 41467_2020_20664_MOESM3_ESM.pdf]

nature research

Corresponding author(s): RUMELA CHAKRABARTI

Last updated by author(s): 01st Dec , 2020

Reporting Summary

Nature Research wishes to improve the reproducibility of the work that we publish. This form provides structure for consistency and transparency in reporting. For further information on Nature Research policies, see Authors & Referees and the Editorial Policy Checklist.

Please do not complete any field with "not applicable" or n/a. Refer to the help text for what text to use if an item is not relevant to your study.
For final submission: please carefully check your responses for accuracy; you will not be able to make changes later.

Statistics

For all statistical analyses, confirm that the following items are present in the figure legend, table legend, main text, or Methods section.

n/a	Confirmed	
☐	☒	The exact sample size (*n*) for each experimental group/condition, given as a discrete number and unit of measurement
☐	☒	A statement on whether measurements were taken from distinct samples or whether the same sample was measured repeatedly
☐	☒	The statistical test(s) used AND whether they are one- or two-sided<br>*Only common tests should be described solely by name; describe more complex techniques in the Methods section.*
☒	☐	A description of all covariates tested
☐	☒	A description of any assumptions or corrections, such as tests of normality and adjustment for multiple comparisons
☐	☒	A full description of the statistical parameters including central tendency (e.g. means) or other basic estimates (e.g. regression coefficient) AND variation (e.g. standard deviation) or associated estimates of uncertainty (e.g. confidence intervals)
☐	☒	For null hypothesis testing, the test statistic (e.g. *F*, *t*, *r*) with confidence intervals, effect sizes, degrees of freedom and *P* value noted<br>*Give P values as exact values whenever suitable.*
☒	☐	For Bayesian analysis, information on the choice of priors and Markov chain Monte Carlo settings
☒	☐	For hierarchical and complex designs, identification of the appropriate level for tests and full reporting of outcomes
☒	☐	Estimates of effect sizes (e.g. Cohen's *d*, Pearson's *r*), indicating how they were calculated

Our web collection on statistics for biologists contains articles on many of the points above.

Software and code

Policy information about availability of computer code

Data collection	FACSDiva software version 5.0.3 (flow cytometry)<br>Nikon [NIS Elements imaging software] version 4.50 (microscopic IHC and IF images)<br>[illegible]
Data analysis	We repeated our in vitro experiments at least three times in triplicate. The exact number of mice for each group is mentioned in the corresponding figure legends. For in vivo treatments of Dll1 blocking antibody, doxorubicin, and IMD, the mice were randomized based on their age and body weight before starting the experiment. Results were reported as mean ± SEM (standard error of the mean). For normally distributed datasets, significance was calculated using a two-tailed Student ' s t-test. The tumor growth datasets were analyzed using the Bonferroni corrected 2-way ANOVA to compute statistical significance. For data with multiple comparisons, 1-way ANOVA with post-hoc TUKEY test was used. The differences between the survival groups of Kaplan-Meier plots were statistically evaluated using Log-Rank tests. For limiting dilution assay, a Chi-square test was used and ELDA online software was used. The number of animals and the statistical tests to compute p-value are reported in each corresponding figure legends.

For manuscripts utilizing custom algorithms or software that are central to the research but not yet described in published literature, software must be made available to editors/reviewers. We strongly encourage code deposition in a community repository (e.g. GitHub). See the Nature Research guidelines for submitting code & software for further information.

Data

Policy information about availability of data

All manuscripts must include a data availability statement. This statement should provide the following information, where applicable:

- Accession codes, unique identifiers, or web links for publicly available datasets
- A list of figures that have associated raw data
- A description of any restrictions on data availability

GSEA Accession codes: GSE129198 and GSE133736.

Field-specific reporting

Please select the one below that is the best fit for your research. If you are not sure, read the appropriate sections before making your selection.

- [x] Life sciences
- [ ] Behavioural & social sciences
- [ ] Ecological, evolutionary & environmental sciences

For a reference copy of the document with all sections, see nature.com/documents/nr-reporting-summary-flat.pdf

Life sciences study design

All studies must disclose on these points even when the disclosure is negative.

Sample size	No statistical methods were used to predetermine the same size. The sample size was chosen by following the literature in the field.
Data exclusions	Animals were excluded only if they died or had to be sacrificed because of moribund conditions specified in the IACUC protocol. Such event occurred to less than 5% of animals during the experimental time frame.
Replication	The in vitro data were reproduced in technical duplicates and experiments were performed at least three times. The exact number of mice used in each group in an experiment is mentioned in corresponding figure legends. All the in vitro experiments were successful.
Randomization	For in vivo treatments of Dll1 blocking antibody, doxorubicin and IMD, the mice were randomized based on their age and body weight, before the start of experiment. Spontaneous tumor mice were not randomized manually. But these mice are obtained from different cages and different litters, so they are automatically randomized.
Blinding	The investigators were not blinded to group allocation or analysis because the same investigator allocated groups and did the analysis. However, the technician and student who performed the measurement of tumor size were blind to the allocation.

Behavioural & social sciences study design

All studies must disclose on these points even when the disclosure is negative.

Study description	*Briefly describe the study type including whether data are quantitative, qualitative, or mixed-methods (e.g. qualitative cross-sectional, quantitative experimental, mixed-methods case study).*
Research sample	*State the research sample (e.g. Harvard university undergraduates, villagers in rural India) and provide relevant demographic information (e.g. age, sex) and indicate whether the sample is representative. Provide a rationale for the study sample chosen. For studies involving existing datasets, please describe the dataset and source.*
Sampling strategy	*Describe the sampling procedure (e.g. random, snowball, stratified, convenience). Describe the statistical methods that were used to predetermine sample size OR if no sample-size calculation was performed, describe how sample sizes were chosen and provide a rationale for why these sample sizes are sufficient. For qualitative data, please indicate whether data saturation was considered, and what criteria were used to decide that no further sampling was needed.*
Data collection	*Provide details about the data collection procedure, including the instruments or devices used to record the data (e.g. pen and paper, computer, eye tracker, video or audio equipment) whether anyone was present besides the participant(s) and the researcher, and whether the researcher was blind to experimental condition and/or the study hypothesis during data collection.*
Timing	*Indicate the start and stop dates of data collection. If there is a gap between collection periods, state the dates for each sample cohort.*
Data exclusions	*If no data were excluded from the analyses, state so OR if data were excluded, provide the exact number of exclusions and the rationale behind them, indicating whether exclusion criteria were pre-established.*
Non-participation	*State how many participants dropped out/declined participation and the reason(s) given OR provide response rate OR state that no participants dropped out/declined participation.*
Randomization	*If participants were not allocated into experimental groups, state so OR describe how participants were allocated to groups, and if allocation was not random, describe how covariates were controlled.*

Ecological, evolutionary & environmental sciences study design

All studies must disclose on these points even when the disclosure is negative.

Study description	*Briefly describe the study. For quantitative data include treatment factors and interactions, design structure (e.g. factorial, nested, hierarchical), nature and number of experimental units and replicates.*
Research sample	*Describe the research sample (e.g. a group of tagged Passer domesticus, all Stenocereus thurberi within Organ Pipe Cactus National Monument), and provide a rationale for the sample choice. When relevant, describe the organism taxa, source, sex, age range and any manipulations. State what population the sample is meant to represent when applicable. For studies involving existing datasets, describe the data and its source.*

Sampling strategy	*Note the sampling procedure. Describe the statistical methods that were used to predetermine sample size OR if no sample-size calculation was performed, describe how sample sizes were chosen and provide a rationale for why these sample sizes are sufficient.*
Data collection	*Describe the data collection procedure, including who recorded the data and how.*
Timing and spatial scale	*Indicate the start and stop dates of data collection, noting the frequency and periodicity of sampling and providing a rationale for these choices. If there is a gap between collection periods, state the dates for each sample cohort. Specify the spatial scale from which the data are taken*
Data exclusions	*If no data were excluded from the analyses, state so OR if data were excluded, describe the exclusions and the rationale behind them, indicating whether exclusion criteria were pre-established.*
Reproducibility	*Describe the measures taken to verify the reproducibility of experimental findings. For each experiment, note whether any attempts to repeat the experiment failed OR state that all attempts to repeat the experiment were successful.*
Randomization	*Describe how samples/organisms/participants were allocated into groups. If allocation was not random, describe how covariates were controlled. If this is not relevant to your study, explain why.*
Blinding	*Describe the extent of blinding used during data acquisition and analysis. If blinding was not possible, describe why OR explain why blinding was not relevant to your study.*

Did the study involve field work? ☐ Yes ☒ No

Field work, collection and transport

Field conditions	*Describe the study conditions for field work, providing relevant parameters (e.g. temperature, rainfall).*
Location	*State the location of the sampling or experiment, providing relevant parameters (e.g. latitude and longitude, elevation, water depth).*
Access and import/export	*Describe the efforts you have made to access habitats and to collect and import/export your samples in a responsible manner and in compliance with local, national and international laws, noting any permits that were obtained (give the name of the issuing authority, the date of issue, and any identifying information).*
Disturbance	*Describe any disturbance caused by the study and how it was minimized.*

Reporting for specific materials, systems and methods

We require information from authors about some types of materials, experimental systems and methods used in many studies. Here, indicate whether each material, system or method listed is relevant to your study. If you are not sure if a list item applies to your research, read the appropriate section before selecting a response.

Materials & experimental systems

n/a	Involved in the study	
☐	☒	Antibodies
☒	☐	Eukaryotic cell lines
☒	☐	Palaeontology
☐	☒	Animals and other organisms
☒	☐	Human research participants
☒	☐	Clinical data

Methods

n/a	Involved in the study	
☒	☐	ChIP-seq
☐	☒	Flow cytometry
☒	☐	MRI-based neuroimaging

Antibodies

Antibodies used

Antibodies used for this study are mentioned below:
For IHC, IF and western blotting analysis:
Anti-Cytokeratin 14, BioLegend, Cat# 905304, RRID:AB_2616896 for IF
Anti-Cytokeratin 14, BioLegend, Cat# 906001, RRID:AB_2565055 for IF
Anti-Cytokeratin 14, Sinobiological, Cat# 100129-RP02, RRID: AB_2860054 for IF
Anti-Cytokeratin K5, Abcam, Cat# ab52635, RRID: AB_869890 for IF
Anti-Cytokeratin K8, TROMA-1s Antibody, Developmental Studies Hybridoma Bank, Cat# AB_531826 for IF
Anti-GFP antibody, AbCam, Cat# ab13970, RRID:AB_300798 for IF
Anti-mCherry Antibody, AbCam Cat# ab167453, RRID:AB_2571870 for IF
Anti-mCherry Antibody, AbCam Cat# ab125096, RRID:AB_11133266 for IF
Mouse Anti-Gamma H2A.X (p S139), Abcam, Cat# ab26350, RRID:AB_470861 for IF
Anti-NF-κβ, Cell Signaling Tech., Cat# 8242 for IHC
Goat anti-Rabbit IgG (H+L) Alexa Fluor 594, Thermo Fisher Scientific, Cat# A-11012, RRID:AB_2534079 for IF

Goat anti-Chicken IgY (H+L), Alexa Fluor 488, Thermo Fisher Scientific, Cat# A-11039, RRID:AB_2534096 for IF
Donkey anti-Rat IgG (H+L), Alexa Fluor 488, Thermo Fisher Scientific, Cat# A-21208, RRID:AB_2535794 for IF
Donkey anti-Rat IgG (H+L), Alexa Fluor 594, Thermo Fisher Scientific, Cat# A-21209, RRID:AB_2535795 for IF

Goat anti-Mouse IgG (H+L), Alexa Fluor 488, Thermo Fisher Scientific, Cat# A-11029, RRID:AB_2534088 for IF
Biotinylated Anti-rabbit IgG (H+L) Antibody, Cat# 176-1506, 1:300 for IHC
Anti-DLL1 Antibody, Abcam, Cat# ab84620, RRID:AB_1860333 for IHC and western blotting
Mouse anti-β-Actin Antibody, Abcam, Cat# ab8227, RRID:AB_2305186 for western blotting
Rabbit IgG HRP Linked Whole Antibody, Cat# NA934, 1:2000 for western blotting

For flow cytometry sorting cells:
PE Rat Anti-mouse CD24, BD Biosciences, Cat# 553262, RRID:AB_394741
BV421 Rat Anti-Mouse CD24, BD Biosciences, Cat# 562563, RRID:AB_2737002
FITC Hamster Anti-Mouse CD29, Bio-Rad, Cat# MCA2298F, RRID:AB_566688
Biotin Rat Anti-CD31, BD Biosciences, Cat# 558737, RRID:AB_397096
Biotin Rat Anti-Mouse CD45, BD Biosciences, Cat#553078, RRID:AB_394608
Biotin Rat Anti-Mouse TER-119, BD Biosciences, Cat#553672, RRID:AB_394985
Rat Anti-Mouse CD29 APC, Bio-Rad, Cat#MCA2298, RRID:AB_566686
For flow cytometry analysis:
BV785 anti-Mouse CD19, BioLegend, Cat#115543, RRID:AB_11218994
APC Anti-Mouse/Human CD45R/B220, BioLegend, Cat#103212, RRID:AB_312997
PE/Cy7 Anti-Mouse CD93, BioLegend, Cat#136506, RRID:AB_2044012
PE Anti-Mouse CD23, BD Biosciences, Cat#553139, RRID:AB_394654
APC/Cy7 Anti-Mouse CD21/35, BioLegend, Cat#123418, RRID:AB_1953275

Antibodies used for in vivo studies
Dll1-blocking antibody, Genentech, 18mg/kg thrice a week for in vivo studies

Commercial Assays
Rabbit Anti-caspase 3, Active; BD Biosciences, Cat#550480, RRID:AB_393697
Click-iT Plus EdU Alexa Fluor 594, Thermo Fisher Scientific, Cat#C10639
Click-iT™ Plus TUNEL Assay for In Situ Apoptosis Detection Kit, Alexa Fluor 488 dye, Thermo Fisher Scientific, Cat#C10617

Validation

1. Anti-Cytokeratin 14, BioLegend, Cat# 905304, RRID:AB_2616896 for IF
The antibody is validated for IF, IHC and western blot using HaCaT cells. More information is available at:
[illegible]

Eukaryotic cell lines

Policy information about cell lines

Cell line source(s)

Not applicable

Authentication

Describe the authentication procedures for each cell line used OR declare that none of the cell lines used were authenticated.

Mycoplasma contamination

Confirm that all cell lines tested negative for mycoplasma contamination OR describe the results of the testing for mycoplasma contamination OR declare that the cell lines were not tested for mycoplasma contamination.

Commonly misidentified lines
(See ICLAC register)

Name any commonly misidentified cell lines used in the study and provide a rationale for their use.

Palaeontology

Specimen provenance

Provide provenance information for specimens and describe permits that were obtained for the work (including the name of the issuing authority, the date of issue, and any identifying information).

Specimen deposition

Indicate where the specimens have been deposited to permit free access by other researchers.

Dating methods

If new dates are provided, describe how they were obtained (e.g. collection, storage, sample pretreatment and measurement), where they were obtained (i.e. lab name), the calibration program and the protocol for quality assurance OR state that no new dates are provided.

☐ Tick this box to confirm that the raw and calibrated dates are available in the paper or in Supplementary Information.

Animals and other organisms

Policy information about studies involving animals; ARRIVE guidelines recommended for reporting animal research

Laboratory animals

Female C57BL/6 mice were used for tumor studies. 4-5 week-old mice were utilized for Dll1+ or Dll1- tumor cells mammary fat
[illegible]

Wild animals

None were used

Field-collected samples

None were used

Ethics oversight

All procedures involving mice and experimental protocols were approved by Institutional Animal Care and Use Committee (IACUC) of University of Pennsylvania.

Note that full information on the approval of the study protocol must also be provided in the manuscript.

Human research participants

Policy information about studies involving human research participants

Population characteristics	*Describe the covariate-relevant population characteristics of the human research participants (e.g. age, gender, genotypic information, past and current diagnosis and treatment categories). If you filled out the behavioural & social sciences study design questions and have nothing to add here, write "See above."*
Recruitment	*Describe how participants were recruited. Outline any potential self-selection bias or other biases that may be present and how these are likely to impact results.*
Ethics oversight	*Identify the organization(s) that approved the study protocol.*

Note that full information on the approval of the study protocol must also be provided in the manuscript.

Clinical data

Policy information about clinical studies

All manuscripts should comply with the ICMJE guidelines for publication of clinical research and a completed CONSORT checklist must be included with all submissions.

Clinical trial registration	N/A
Study protocol	N/A
Data collection	Data is collected from published online freely available database (KM plotter)
Outcomes	N/A

ChIP-seq

Data deposition

☐ Confirm that both raw and final processed data have been deposited in a public database such as GEO.

☐ Confirm that you have deposited or provided access to graph files (e.g. BED files) for the called peaks.

Data access links<br>*May remain private before publication.*	*For "Initial submission" or "Revised version" documents, provide reviewer access links. For your "Final submission" document, provide a link to the deposited data.*
Files in database submission	*Provide a list of all files available in the database submission.*
Genome browser session<br>(e.g. UCSC)	*Provide a link to an anonymized genome browser session for "Initial submission" and "Revised version" documents only, to enable peer review. Write "no longer applicable" for "Final submission" documents.*

Methodology

Replicates	*Describe the experimental replicates, specifying number, type and replicate agreement.*
Sequencing depth	*Describe the sequencing depth for each experiment, providing the total number of reads, uniquely mapped reads, length of reads and whether they were paired- or single-end.*
Antibodies	*Describe the antibodies used for the ChIP-seq experiments; as applicable, provide supplier name, catalog number, clone name, and lot number.*
Peak calling parameters	*Specify the command line program and parameters used for read mapping and peak calling, including the ChIP, control and index files used.*
Data quality	*Describe the methods used to ensure data quality in full detail, including how many peaks are at FDR 5% and above 5-fold enrichment.*
Software	*Describe the software used to collect and analyze the ChIP-seq data. For custom code that has been deposited into a community repository, provide accession details.*

Flow Cytometry

Plots

Confirm that:

☑ The axis labels state the marker and fluorochrome used (e.g. CD4-FITC).

Methodology

Sample preparation	Tumors were processed to obtain single cell suspension as described previously by Chakrabarti et al., 2014. Single cells were incubated with combination of antibodies as per experimental requirement for 30 min in dark.
Instrument	Aria II instrument was used for sorting. LSRII and LSR Fortessa Flow Cytometer instruments were used for cell population analyses (100 uM nozzle was used and 20 psi pressure was applied).
Software	FACS Vantage (Aria II and FACS Jazz) and FlowJo software v10 (TreeStar, Inc).
Cell population abundance	Dll1+(mCherry+) tumor cell population ranged from 50-75% in MMTV-PyMT;Dll1-mCherry tumors.
Gating strategy	Gating strategies are clearly described in the figure legends. Basically, live cells were gated with FCS and SSC and subsequently gated with various markers as indicated in the figures.

☑ Tick this box to confirm that a figure exemplifying the gating strategy is provided in the Supplementary Information.

Magnetic resonance imaging

Experimental design

Design type	*Indicate task or resting state; event-related or block design.*
Design specifications	*Specify the number of blocks, trials or experimental units per session and/or subject, and specify the length of each trial or block (if trials are blocked) and interval between trials.*
Behavioral performance measures	*State number and/or type of variables recorded (e.g. correct button press, response time) and what statistics were used to establish that the subjects were performing the task as expected (e.g. mean, range, and/or standard deviation across subjects).*

Acquisition

Imaging type(s)	*Specify: functional, structural, diffusion, perfusion.*
Field strength	*Specify in Tesla*
Sequence & imaging parameters	*Specify the pulse sequence type (gradient echo, spin echo, etc.), imaging type (EPI, spiral, etc.), field of view, matrix size, slice thickness, orientation and TE/TR/flip angle.*
Area of acquisition	*State whether a whole brain scan was used OR define the area of acquisition, describing how the region was determined.*

Diffusion MRI ☐ Used ☐ Not used

Preprocessing

Preprocessing software	*Provide detail on software version and revision number and on specific parameters (model/functions, brain extraction, segmentation, smoothing kernel size, etc.).*
Normalization	*If data were normalized/standardized, describe the approach(es): specify linear or non-linear and define image types used for transformation OR indicate that data were not normalized and explain rationale for lack of normalization.*
Normalization template	*Describe the template used for normalization/transformation, specifying subject space or group standardized space (e.g. original Talairach, MNI305, ICBM152) OR indicate that the data were not normalized.*
Noise and artifact removal	*Describe your procedure(s) for artifact and structured noise removal, specifying motion parameters, tissue signals and physiological signals (heart rate, respiration).*
Volume censoring	*Define your software and/or method and criteria for volume censoring, and state the extent of such censoring.*

Statistical modeling & inference

Model type and settings	*Specify type (mass univariate, multivariate, RSA, predictive, etc.) and describe essential details of the model at the first and second levels (e.g. fixed, random or mixed effects; drift or auto-correlation).*
Effect(s) tested	*Define precise effect in terms of the task or stimulus conditions instead of psychological concepts and indicate whether ANOVA or factorial designs were used.*

Specify type of analysis: ☐ Whole brain ☐ ROI-based ☐ Both

Statistic type for inference

(See Eklund et al. 2016)

Specify voxel-wise or cluster-wise and report all relevant parameters for cluster-wise methods.

Correction

Describe the type of correction and how it is obtained for multiple comparisons (e.g. FWE, FDR, permutation or Monte Carlo).

Models & analysis

n/a	Involved in the study
☐	☐ Functional and/or effective connectivity
☐	☐ Graph analysis
☐	☐ Multivariate modeling or predictive analysis

Functional and/or effective connectivity

Report the measures of dependence used and the model details (e.g. Pearson correlation, partial correlation, mutual information).

Graph analysis

Report the dependent variable and connectivity measure, specifying weighted graph or binarized graph, subject- or group-level, and the global and/or node summaries used (e.g. clustering coefficient, efficiency, etc.).

Multivariate modeling and predictive analysis

Specify independent variables, features extraction and dimension reduction, model, training and evaluation metrics.

This checklist template is licensed under a Creative Commons Attribution 4.0 International License, which permits use, sharing, adaptation, distribution and reproduction in any medium or format, as long as you give appropriate credit to the original author(s) and the source, provide a link to the Creative Commons license, and indicate if changes were made. The images or other third party material in this article are included in the article's Creative Commons license, unless indicated otherwise in a credit line to the material. If material is not included in the article's Creative Commons license and your intended use is not permitted by statutory regulation or exceeds the permitted use, you will need to obtain permission directly from the copyright holder. To view a copy of this license, visit http://creativecommons.org/licenses/by/4.0/
